# *TRIM8*-associated non-coding RNA panel as a biomarker for Lupus nephritis activity

**DOI:** 10.1186/s12967-025-07137-3

**Published:** 2025-11-05

**Authors:** Mostafa Abdelnasier Abd Elgawad, Howayda Abdelhamid El Shinnawy, Sanaa Eissa, Nouran Abdelfattah Sayed Ali, Maha Abdelmoneim Behairy, Cherry Reda Kamel, Marwa Mostafa Kamel

**Affiliations:** 1 Internal Medicine and Nephrology Department, Faculty of Medicine, Ain Shams University, Abbassia, Cairo 11566 Egypt; 2 Medical Biochemistry and Molecular Biology Department, Faculty of Medicine, Ain Shams University, Abbassia, Cairo 11381 Egypt

**Keywords:** Lupus nephritis, *TRIM8*, *hsa-miR-126-5p*, *lnc-SSBP2-1:1*, non-coding RNA network, Biomarkers, Disease activity

## Abstract

**Background:**

Lupus nephritis (LN) represents a major complication in systemic lupus erythematosus (SLE). The objective of this study was to evaluate the *TRIM8* gene and its associated non-coding RNAs (*lnc-SSBP2-1:1* and *hsa-miR-126-5p*) as potential non-invasive biomarkers for LN activity.

**Methods:**

Bioinformatics analyses were initially employed to identify candidate mRNA and associated non-coding RNAs (ncRNAs) implicated in LN. Expression profiles of *TRIM8lnc-SSBP2-1:1*and *hsa-miR-126-5p* were validated in blood samples from 40 active LN, 30 inactive LN patients, and 20 healthy individuals via real-time PCR.

**Results:**

*TRIM8* mRNA and *lnc-SSBP2-1:1* lncRNA levels were notably upregulated in active LN (*p* < 0.001), while *hsa-miR-126-5p *was reduced (*p* < 0.001). SLEDAI-2K scores correlated positively with *TRIM8* mRNA and *lnc-SSBP2-1:1*, and negatively with *hsa-miR-126-5p*.

**Conclusions:**

This study highlights *TRIM8*-associated ncRNA regulatory network as promising biomarkers in LN activωity, with potential clinical impact.

**Graphical abstract:**

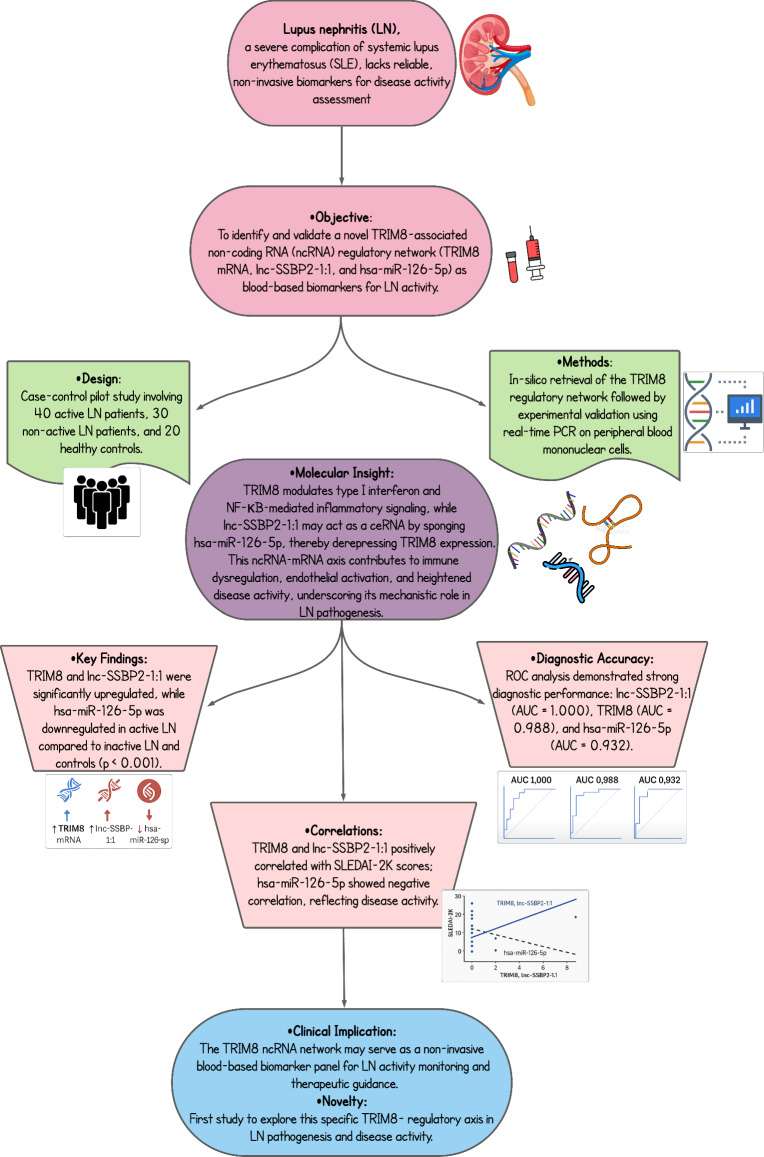

**Electronic supplementary material:**

The online version of this article (10.1186/s12967-025-07137-3) contains supplementary material, which is available to authorized users.

## Highlights

- *TRIM8* and *lnc-SSBP2-1:1* are upregulated, *hsa-miR-126-5p* is downregulated in active LN
- Biomarkers strongly correlated with SLEDAI-2K disease activity scores
- High diagnostic accuracy (AUCs > 0.93) in distinguishing active vs inactive LN
- Potential non-invasive tool to complement current LN monitoring

**Electronic supplementary material:**

The online version of this article (10.1186/s12967-025-07137-3) contains supplementary material, which is available to authorized users.

## Background

Lupus nephritis (LN) is known to be one of the most severe complications in systemic lupus erythematosus (SLE), affecting up to seventy percent of the individuals diagnosed with the disease. [1]. Despite existing treatments, about 10% of patients advance to end-stage kidney disease (ESKD), posing a major clinical and socioeconomic burden [2]. Traditional biomarkers, such as anti-dsDNA antibodies and complement (C3/C4), often lack sensitivity and specificity in tracking disease activity or predicting flares. Thus, identifying reliable non-invasive markers [3].

The tripartite motif-containing protein 8 (*TRIM8*), a member of the tripartite motif family, is a key regulator of immune responses through NF-κB and type-I interferon signaling [4–6]. Its role in IFN-γ hyper-responsiveness has been implicated in autoimmune conditions and renal inflammation [7, 8], suggesting relevance in LN pathogenesis. dysregulation of non-coding RNAs modulates these pathways and correlates with disease severity [9–12]. However, the role of *TRIM8*-associated ncRNA network in LN activity remains unexplored.

This study aimed to evaluate the expression of *TRIM8* and its associated ncRNAs (*lnc-SSBP2-1:1 and hsa-miR-126-5p*) as potential biomarkers for LN activity and disease severity.

## Patients and methods

Our study is a case–control pilot study including 40 active LN, 30 inactive LN, and 20 healthy controls at, internal medicine department of Ain Shams University Hospitals. All 70 patients fulfilled the 2019 EULAR/ACR classification criteria and had a biopsy confirming LN at the time of diagnosis [13].Active LN defined by proteinuria > 0.5 g/24h, active urinary sediments, or biopsy evidence. Inactive LN defined as remission with proteinuria < 0.5 g/24h.

Patients with diabetes, uncontrolled hypertension, ischemic heart disease, active infection, decompensated liver disease, malignancy, other auto-immune diseases, pregnancy, or on renal replacement therapy were excluded. Ethical approval was provided from Ain Shams University REC (FWA 000017585). Informed consent obtained.

### Demographic and clinical data

Disease activity was scored using the systemic lupus erythematosus disease activity index 2000 (SLEDAI-2K) score, from zero activity, scores 1–5 mild activity, 6–10 moderate activity, 11–19 high activity, and scores exceeding 20 points are very high activity.

Additionally, the renal component of the Systemic Lupus Erythematosus Disease Activity Index (rSLEDAI) was applied to assess the renal involvement, it considers four renal parameters encompassing hematuria, pyuria, proteinuria, and urinary casts. Every one of these indicators adds four points to the total score, ranging from zero (signifying inactive renal disease) to 16 (representing the highest level of disease activity) [14].

### Laboratory investigations

Included CBC, creatinine, eGFR (CKD-EPI 2021), complements, ANA, anti-dsDNA, CRP, protein/creatinine ratio, and urine microscopy [15].

### Bioinformatics-based identification of novel genetic and epigenetic regulatory networks

Genetic network selection: initially, relevant molecular pathways associated with LN pathogenesis were identified using publicly available databases, including NCBI (https://www.ncbi.nlm.nih.gov/) and Reactome (https://reactome.org/). Next, we used these databases to retrieve novel genes related to the INF stimulator gene pathway (cGAS-STING Pathway), a highly implicated recent pathway in the pathogenesis of Lupus Nephritis. Filtering results identified the *TRIM8* gene to be related to the Interferon response in LN. We verified the expression of the selected gene in relevant tissues (kidney and blood) using Human Protein Atlas database (https://www.proteinatlas.org/) and then verified gene ontology (function) from Uniprot database (https://www.uniprot.org/), to make sure it was involved in the disease pathogenesis. Finally, checked the novelty of the selected gene in lupus using PubMed (https://pubmed.ncbi.nlm.nih.gov/) as shown in Supp. Figure 1.

Selection of epigenetic regulators: Retrieval of novel miRNA and lncRNA related to the selected gene to complete the genetic regulatory network, where *hsa-miR-126-5p* was chosen from the mirDB database (https://mirdb.org/) and then confirmed the selected miRNA from another database miRBase database (https://www.mirbase.org/). Finally, the long non-coding RNA *lnc-SSBP2-1:1* was selected based on its predicted interaction with *miR-126-5p*, as identified using the miRWalk2.0 database (http://mirwalk.umm.uni-heidelberg.de/), and the selection was subsequently confirmed using the LNCipedia database (https://lncipedia.org/). At last, we checked the novelty of both selected epigenetic regulatory networks (lncRNA and miRNA) using PubMed, as shown in Supp. Figure 2.

### Validation of the novel TRIM8-associated ncRNA regulatory network in patients’ samples by molecular assay

#### Sample processing

Isolation of Peripheral blood mononuclear cells (PBMNCs) from EDTA-anticoagulated venous blood using the Lymphoprep™ (Axis-Shield PoC AS, Oslo, Norway). Blood samples were diluted with 0.9% NaCl and gently layered over Lymphoprep™, then underwent centrifugation at 800 × g at room temperature for 20 min. After centrifugation, the mononuclear cell layer at the interface was carefully collected. Plasma was removed, and white blood cells were transferred to a clean tube, diluted again with saline, and centrifuged at 250 × g for 10 min to pellet the cells. This protocol ensured effective PBMNC isolation for downstream analyses. Given the limitations of renal biopsy, blood-based biomarkers offer a promising non-invasive alternative for monitoring immune-mediated diseases [16, 17].

### Quantification of RNA expression using RT-qPCR

#### Total RNA extraction

Total RNA, including lncRNA and miRNA, was extracted from PBMNCs using the miRNeasy Mini Kit (Qiagen, Cat. No. 217004) following the protocol of the manufacturer. A thermoscientific nanodrop spectrophotometer, with purity ratios ranging between 1.8 and 2.0, was used to assess RNA concentration and integrity.

#### Reverse transcription to cDNA

RNA templates were reverse transcribed into cDNA using the RT^2^ First Strand Kit (Qiagen, Cat. No. 330404) for mRNA and lncRNA, and the miRCURY LNA miRNA PCR Starter Kit (Qiagen, Cat. No. 339320) for miRNA. This reverse transcription process was conducted using the Thermo Hybrid PCR Express Thermal Cycler (Thermo Fisher, USA), in strict adherence to the manufacturer’s instructions.

#### Quantification of TRIM8-associated ncRNA regulatory network expression by RT-qPCR

An RT2 SYBR Green qPCR Mastermix Kit (Qiagen, Cat. No. 330502) and QuantiTect Primer Assays for *TRIM-8* (Hs_TRIM8_1_SG QuantiTect Primer Assay) and *Inc-SSBP2-1:1* (Hs_SSBP2_1_SG QuantiTect Primer Assay) (Qiagen, Cat. No. 249900) were utilized to quantify the mRNA expression of *TRIM-8* and *Inc-SSBP2* in peripheral blood mononuclear cells (PBMNCs), respectively. The (Hs_ACTB_1_SG QuantiTect Primer Assay) (Qiagen, Cat. No.6 249,900) served as the reference assay. To assess *miR-126-5p* expression in all samples, a miRCURY LNA SYBR PCR Starter Kit (Qiagen, Cat. No. 339320) was used along with a miRNA Primer Assay targeting mature *miR-126-5p* (MIMAT0000444). The miRNA Primer Assay targeting mature hsa-miR-103a-3p (MIMAT0000101) was used as an endogenous control assay, following the protocol of the manufacturer.

Quantitative PCR was performed using SYBR Green dye on the Applied Biosystems 7500 Real-Time PCR System (USA). The thermal cycling protocol included an initial 15 min denaturation at 95 °C, followed by 40 cycles of 10 s denaturation at 94 °C and 30 s annealing at 55 °C, with a 30 s final extension at 70 °C. Reactions were run in duplicate. CT values above 36 were considered negative. The 2^− ΔΔCt^ method was employed in determining the relative RNA expression levels, and melting curve analysis was conducted to verify amplification specificity [18].

### Statistical analysis

Data were analyzed using IBM SPSS v28. Normality was tested with the Shapiro–Wilk and Kolmogorov–Smirnov tests. Parametric data are expressed as mean ± SD and analyzed using t-tests or ANOVA. Non-parametric data were evaluated with the Mann–Whitney test, and correlations were assessed using Pearson or Spearman methods based on distribution. Categorical variables were analyzed with Chi-square or Fisher’s Exact tests, applying Bonferroni correction when needed. ROC curve analysis determined the diagnostic performance of the studied markers, with statistical significance set at *p* ≤ 0.05.

## Results

### Demographics and disease characteristics

Gender distribution was similar across groups, with females representing 70% of the active LN group, 86.7% of the non-active LN group, and 75% of the control group. Mean age was 26.7 ± 8.2 years in the active LN group, 28.6 ± 6.9 years in the non-active group, and 29.0 ± 4.0 years in controls, without a significant difference. Most of the active LN patients were class IV (52.5%) and class III (37%), while non-active LN patients were 50% class III, 23% class IV, 13.3% class II, and 13.3% class V in renal biopsies taken at the initial time of diagnosis before the start of therapy. The active LN group had a significantly shorter SLE disease duration (*p* < 0.001), a higher prevalence of hypertension (*p* = 0.013), as well as a significantly higher renal SLEDAI and SLEDAI 2K scores (*p* < 0.001), as shown in Supp. Table 1.

### Laboratory findings

In the active LN group, about 72.5% were anti-DNA positive. Statistically significant differences were found between the active LN and the non-active LN groups regarding the serum albumin level (mean ± SD 2.4 ± 0.5 vs 3.6 ± 0.3), C3 (mean ± SD 55.1 ± 24.8 vs 90.0 ± 21.0), and C4 (mean ± SD 9.0 ± 5.5 vs 21.3 ± 8.7). Serum albumin levels, C3, and C4 were significantly lower in the active LN group compared to the non-active group (*p* < 0.001), with lower eGFR (mean ± SD 71.2 ± 48.6 vs 96.6 ± 32.1) (*p* = 0.011). Also, results of the current study demonstrated significant differences between the active and non-active LN groups regarding serum creatinine level (mean ± SD 1.9 ± 1.5 vs 0.9 ± 0.3), serum BUN (mean ± SD 44.5 ± 30.1 vs 21.5 ± 8.3), Pr/Cr ratio (mean ± SD 3.5 ± 1.6 vs 0.3 ± 0.1) (*p* < 0.001). There were statistically significant differences between active and non-active LN regarding other studied parameters, including hemoglobin, and urine analysis of active sediments (*p* < 0.001). WBC count, platelets, CRP, or ANA titer showed no significant differences as shown in Table 1.

**Table 1:** Comparison between the active and the non-active LN groups with regard to laboratory data

Variables (Mean ± SD)	Active lupus nephritis (Total = 40)	Non-active lupus nephritis (Total = 30)	*p*-value
Hemoglobin (gm/dL)	9.3 ± 2.2	11.4 ± 1.9	^ < 0.001*
WBC (× 10^3^/mL)	6.7 ± 2.6	6.9 ± 3.0	^0.708
Platelets (× 10^3^/mL)	257.5 ± 96.8	246.9 ± 92.3	^0.649
Serum albumin (gm/dL)	2.4 ± 0.5	3.6 ± 0.3	^ < 0.001*
Serum creatinine (mg/dL)	1.9 ± 1.5	0.9 ± 0.3	^ < 0.001*
Serum BUN (mg/dL)	44.5 ± 30.1	21.5 ± 8.3	^ < 0.001*
eGFR (mL/min/1.73m^2^)	71.2 ± 48.6	96.6 ± 32.1	^0.011*
CRP (mg/dL)	5.4 ± 3.4	4.6 ± 2.9	^0.343
C3 (mg/dL)	55.1 ± 24.8	90.0 ± 21.0	^ < 0.001*
C4 (mg/dL)	9.0 ± 5.5	21.3 ± 8.7	^ < 0.001*
ANA titer	115.9 ± 61.4	96.0 ± 73.2	^0.224
Anti-DNA	29 (72.5%)	0 (0.0%)	# < 0.001*
Pr/Cr ratio (gm/gm)	3.5 ± 1.6	0.3 ± 0.1	^ < 0.001*
Pus cells in urine (/HPF)	25 (62.5%)	0 (0.0%)	# < 0.001*
RBCs in urine (/HPF)	19 (47.5%)	0 (0.0%)	# < 0.001*
Urinary casts (/HPF)	16 (40.0%)	0 (0.0%)	# < 0.001*

^*^ Significant *p*-value < 0.05. Mean ± SD. ^ Independent t-test. # Chi-square test

### Validation of the novel genetic and epigenetic network in patients’ sample

**Fig. 1:**
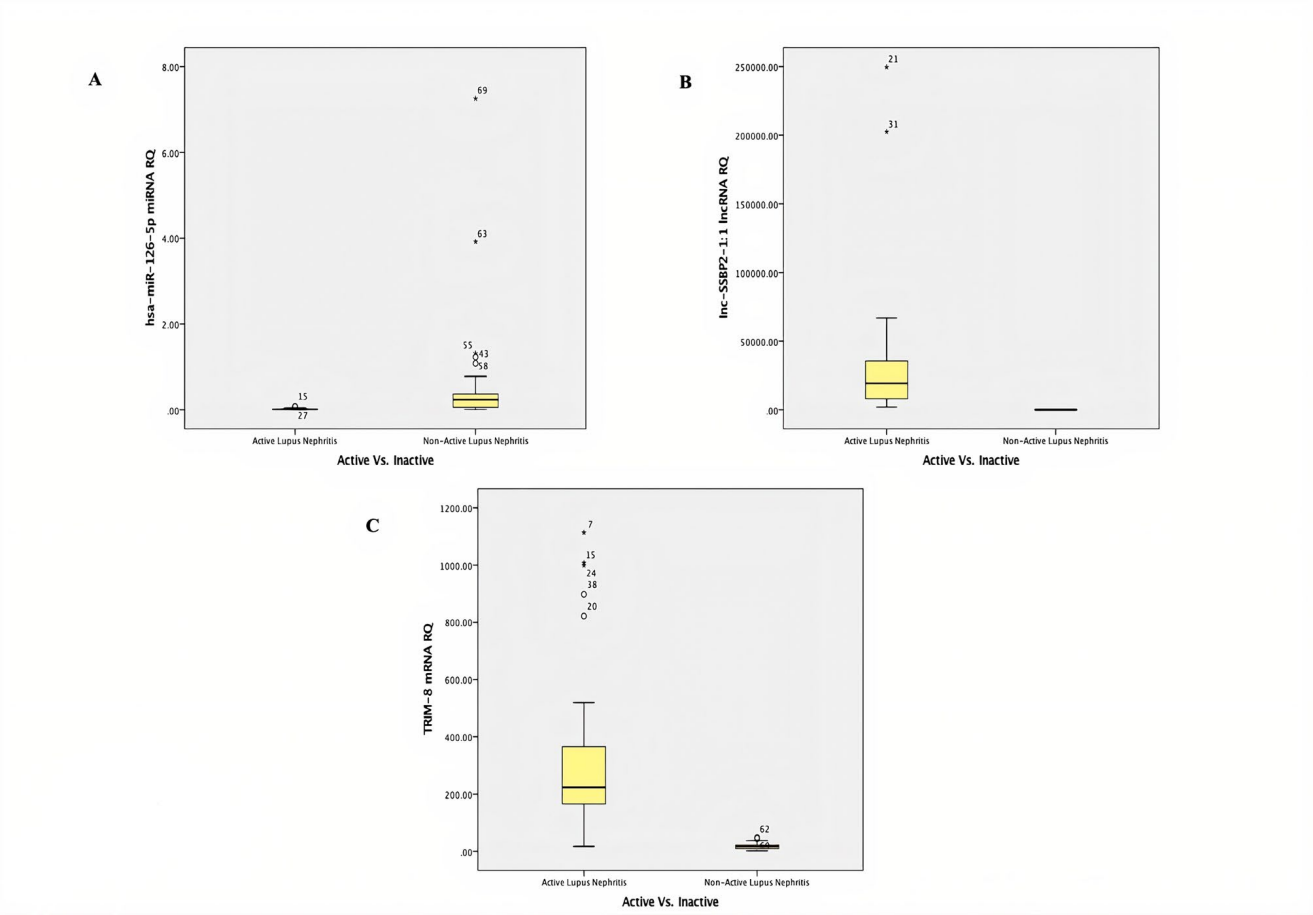
Boxplots showing differential expression (RQ) of the *TRIM8*-associated ncRNA regulatory network in active vs. inactive lupus nephritis (LN) patients measured by qRT-PCR: **A** *hsa-miR-126-5p* miRNA (*p* < 0.001), **B** *lnc-SSBP2-1:1* lncRNA (*p* < 0.001), **C** *TRIM8* mRNA (*p* < 0.001). Boxes represent the interquartile range (IQR), horizontal lines represent the median, and whiskers indicate 1.5 × IQR. Circles denote outliers. Group differences were analyzed using the Mann–Whitney U test

**Fig. 2:**
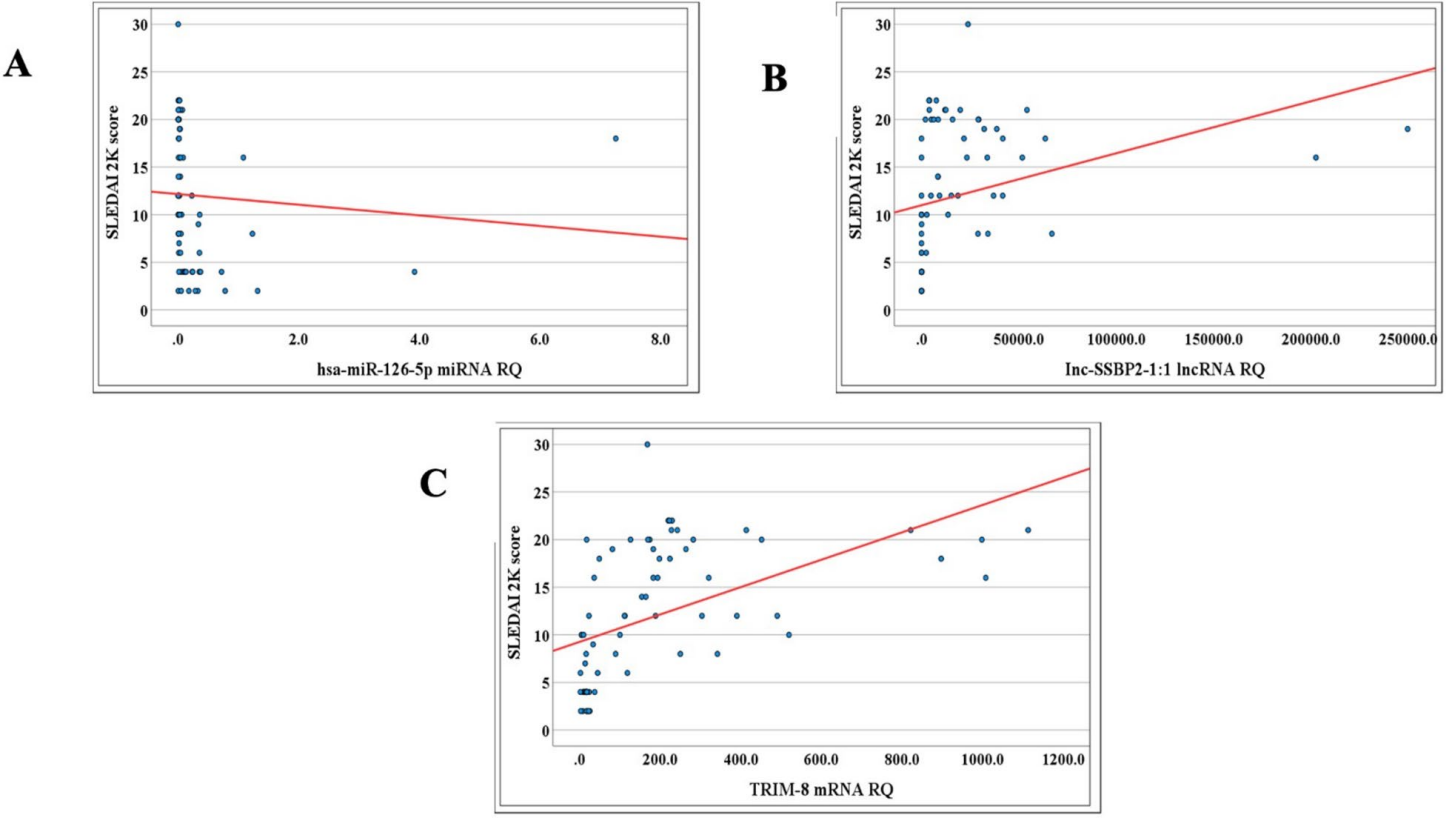
Correlation between disease activity (SLEDAI-2K score) and expression levels of **A*** hsa-miR-126-5p* (r = − 0.517, *p* < 0.001), **B** *lnc-SSBP2-1:1* (r =  + 0.600, *p* < 0.001), and **C** *TRIM8* mRNA (r =  + 0.692, *p* < 0.001). Correlation analysis was performed using Spearman’s rho test. Red lines represent regression fit

The mean ranks of RQ of *TRIM8* mRNA and *lnc-SSBP2-1:1* lncRNA expression levels were significantly higher in the active LN group, whereas *hsa-miR-126-5p* expression was notably reduced compared to the non-active group (*p* < 0.001) as illustrated in Table 2 and Fig. 1. Significant positive correlations were found between global SLEDAI 2K scores and RQ of *TRIM8* mRNA (r = 0.692, *p* = 0.001) and *lnc-SSBP2-1:1* lncRNA expression (r = 0.600, *p* = 0.001), while *hsa-miR-126-5p* expression had a significant negative correlation (r =  − 0.517, *p* = 0.001) as shown in Table 3 and Fig. 2.In the active LN group, RQ of *hsa-miR-126-5p* expression levels showed a negative correlation to serum albumin (r =− 0.521, *p* = 0.001), and *lnc-SSBP2-1:1* expression level had a negative correlation to the duration of the disease (r = − 0.353, *p* = 0.026). RQ of *TRIM8* mRNA and *hsa-miR-126-5p* expression levels were elevated in patients with positive anti-DNA antibodies (*p* = 0.013, *p* = 0.038, respectively) as shown in Supp. Table 2. In the non-active LN group, RQ of *TRIM8* mRNA expression was significantly elevated among hypertensive patients (mean ± SD 32.3 ± 15.0) (*p* = 0.002), while *lnc-SSBP2-1:1* expression showed a significant inverse correlation with ANA titer (r = − 0.437, *p* = 0.016) as shown in Supp. Tables 3 and 4.

**Table 2:** Comparison between the active and non-active LN groups regarding the relative quantification (RQ) of the *TRIM8*-associated ncRNA regulatory network (*hsa-miR-126-5p* and *lnc-SSBP2-1:1*) expressions

Mean rank of RQ of the studied genes	Active lupus nephritis (Total = 40)	Non-active lupus nephritis (Total = 30)	U	*p*-value
*TRIM8* mRNA RQ	50.13	16.00	15.00	< 0.001*
*hsa-miR-126-5p* miRNA RQ	22.54	52.78	81.50	< 0.001*
*lnc-SSBP2-1:1* lncRNA RQ	50.50	15.50	0.00	< 0.001*

Mann–Whitney Test. *Significant

**Table 3:** Correlations between RQ of *TRIM8*-associated ncRNA regulatory network expressions and SLEDAI 2K global activity score among SLE patients

RQ of the studied genes	SLEDAI 2K score	
	R	*p*-value
*TRIM8* mRNA RQ	0.692	< 0.001*
*hsa-miR-126-5p* miRNA RQ	− 0.517	< 0.001*
*lnc-SSBP2-1:1* lncRNA RQ	0.600	< 0.001*

Total = 70, Spearman test. *Significant

**Fig. 3:**
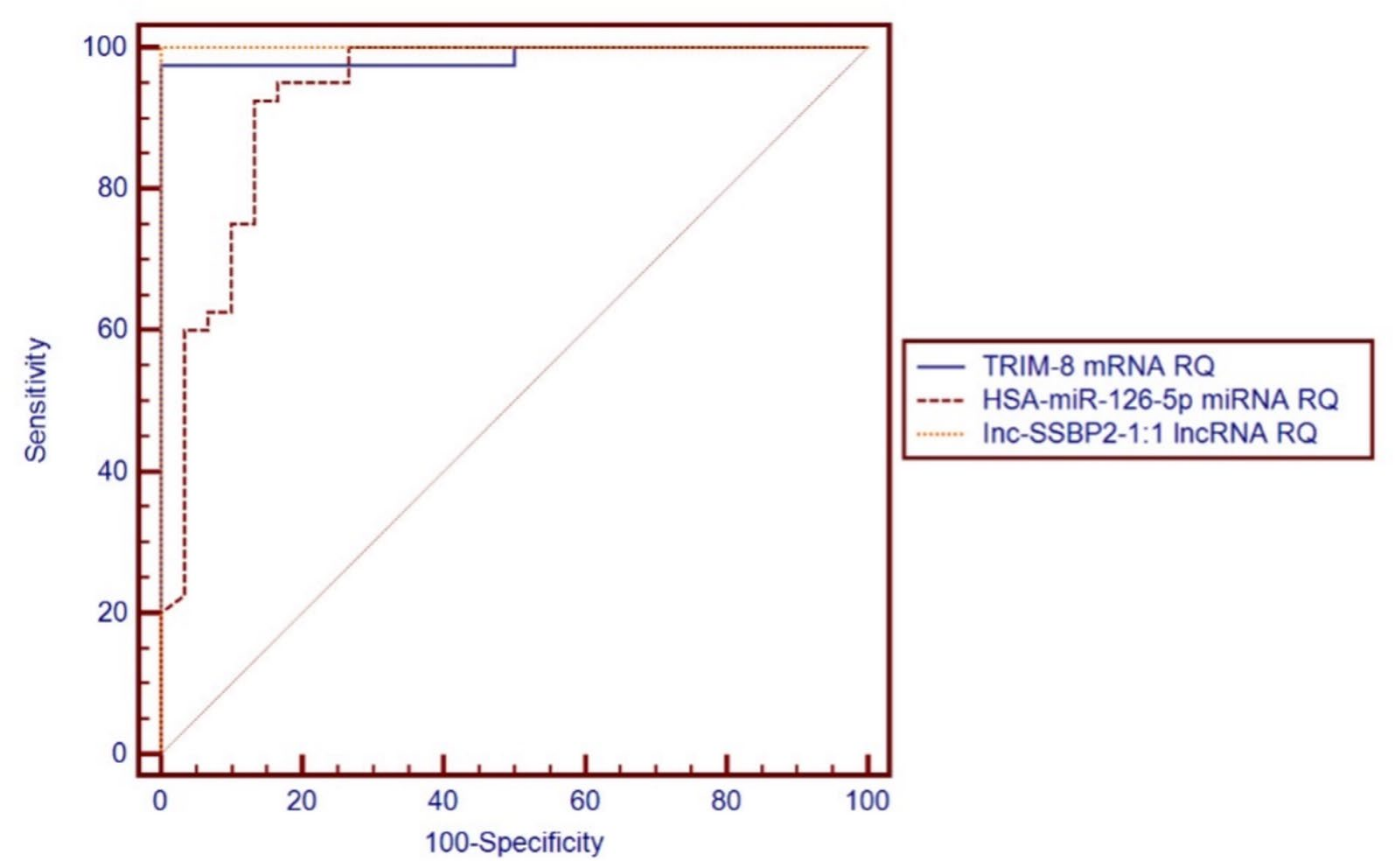
Receiver operating characteristic (ROC) curves of *TRIM8*-associated ncRNA regulatory 548 network expressions for distinguishing active from inactive LN patients. Area under the curve 549 (AUC) values: *TRIM8 *mRNA = 0.988 (95% CI 0.963–1.000, SE = 0.013, *p* < 0.001); *hsa-miR-*550 *126-5p *miRNA = 0.932 (95% CI 0.867–0.997, SE = 0.033, *p* < 0.001); *lnc-SSBP2-1:1 *lncRNA = 551 1.000 (95% CI 1.000–1.000, SE = 0.000, *p* < 0.001)

The ROC curve analysis for the relative quantification (RQ) of *TRIM8*-associated ncRNA regulatory network markers revealed significant discriminatory power between active and non-active lupus nephritis patients. *lnc-SSBP2-1:1* had an AUC of 1.000 (95% CI 1.000–1.000; *p* < 0.001) at a cut—off ≥ 478.63; *TRIM8* had an AUC of 0.988 (95% CI 0.963–1.000; *p* < 0.001) at a cut—off ≥ 63.10; and *hsa-miR-126-5p* yielded an AUC of 0.932 (95% CI 0.867–0.997; *p* < 0.001) at a cut-off ≤ 0.07, as shown in Table 4 and Fig. 3.

**Table 4:** Diagnostic performance of RQ of *TRIM8*-associated ncRNA regulatory network expressions in discriminating the active from the non-active LN groups:

RQ of the studied Genes	AUC	SE	*p*-value	95% CI	Cut-off point
*TRIM8* mRNA RQ	0.988	0.013	< 0.001*	0.963–1.000	≥ 63.10
*hsa-miR-126-5p* miRNA RQ	0.932	0.033	< 0.001*	0.867–0.997	≤ 0.07
*lnc-SSBP2-1:1* lncRNA RQ	1.000	0.000	< 0.001*	1.000–1.000	≥ 478.63

Total = 70, *significant *p*-value < 0.05; AUC, Area under the curve; SE, standard error; CI, confidence interval

Additionally, ROC curve analysis of traditional biomarkers (serum creatinine, C3, and C4) showed AUC values of 0.329 (*p* = 0.015), 0.872 (*p* < 0.001), and 0.889 (*p* < 0.001), respectively. While C3 and C4 showed some discrimination, their limited specificity, along with the poor performance of creatinine, highlights the inadequacy of traditional markers for accurate LN monitoring (Supp. Figure 3).

The statistical analysis showed that *lnc-SSBP2-1:1* had the highest diagnostic accuracy for distinguishing active from non-active LN, achieving 100% sensitivity and specificity. This was followed by *TRIM8* mRNA with 97.5% sensitivity and 100% specificity, and *hsa-miR-126-5p* with 92.5% sensitivity and 86.7% specificity, as shown in Table 5.

**Table 5:** Performance characteristics of the RQ of *TRIM8-*associated ncRNA regulatory network expressions using the calculated cut-off points in discriminating the active LN group from the non-active LN group:

Characteristics	*TRIM-8* mRNA RQ ≥ 63.10 (%)	*hsa-miR-126-5p* miRNA (%) RQ ≤ -0.07	*Inc-SSBP2-1:1* lncRNA (%) RQ ≥ 478.63
Sensitivity	97.5 (86.8–99.9%)	92.5 (79.6–98.4%)	100.0 (91.2–100.0%)
Specificity	100.0 (88.4–100.0%)	86.7 (69.3–96.2%)	100.0 (88.4–100.0%)
Diagnostic accuracy	98.6 (92.3–100.0%)	90.0 (80.5–95.9%)	100.0 (94.9–100.0%)
Youden’s index	97.5 (92.7–100.0%)	79.2 (64.5–93.8%)	100.0 (100.0–100.0%)
Positive predictive value	100.0 (91.0–100.0%)	90.2 (76.9–97.3%)	100.0 (91.2–100.0%)
Negative predictive value	96.8 (83.3–99.9%)	89.7 (72.6–97.8%)	100.0 (88.4–100.0%)

Data expressed as values (95% confidence interval)

## Discussion

Lupus nephritis, a severe complication affecting up to 70% of SLE patients, significantly contributes to morbidity, ESKD, and mortality despite advances in immunosuppressive therapies [19]. Recent data indicate that 10–20% of LN patients still progress to ESKD within 10 years, reflecting limitations of current monitoring and treatment strategies [20, 21]. The present study is among the first to identify and validate a *TRIM8*-associated ncRNA regulatory network in LN, showing that *TRIM8* mRNA and *lnc-SSBP2-1:1* are significantly upregulated, while *hsa-miR-126-5p* is downregulated in active disease.

Although these results are encouraging, it is still unclear how specific this biomarker panel is to LN. Current evidence supports plausibility but not exclusivity. TRIM8 is a ubiquitin E3 ligase that promoting TAK1 Lys63-ubiquitination (NF-κB) and relieving STAT inhibition via PIAS3 degradation (JAK/STAT) [5, 22], so elevated *TRIM8* in active LN is biologically credible, but *TRIM8* dysregulation can also occur in other autoimmune conditions such as OA and keratitis [23, 24] and non-autoimmune glomerulonephritis [25].

*miR-126-5p* is classically endothelial/immune-regulatory; its levels shift in vascular and renal injury across diseases as CKD [26–28]. Its downregulation in active LN may reflect endothelial dysfunction in inflamed glomeruli [29–31]. *lnc-SSBP2-1:1* upregulation indicates a common mechanism for AS-lncRNAs, which is antisense lncRNA–sense gene regulatory coupling (chromatin/accessibility, transcriptional interference). This locus’s functional significance in immunity is conceivable but not yet established [32, 33]. Panel-level performance (*TRIM8* + *lnc-SSBP2-1:1* + *miR-126-5p*) is what may confer relative specificity to active LN versus quiescent SLE as shown in the current data.

A key player in LN pathogenesis, TRIM8 is a crucial regulator of type-I interferon (IFN-I) and NF-κB signaling [5, 6, 24]. TRIM8 increases immune complex-mediated kidney damage by stimulating the production of pro-inflammatory cytokines (TNF-α, IL-6, and IFN-γ) [24, 25]. Prior research has supported TRIM8’s significance in IFN-γ hyper-responsiveness by connecting its deregulation to renal cell cancer [7] and macrophage activation in systemic juvenile idiopathic arthritis [8]. Our results indicate *TRIM8* as a potential therapeutic target and extend this evidence to LN. Its expression positively correlated with SLEDAI-2K scores, highlighting its potential as a disease activity biomarker.

Known for its function in inflammation and endothelial homeostasis [13, 29], *hsa-miR-126-5p* was significantly downregulated in active LN in our investigation. This decrease suggests a wider role in inflammatory renal disease and is consistent with observations of *miR-126* downregulation in sepsis-associated AKI [31] and OA [24]. In our population, its inverse relationship with serum albumin might be a reflection of the degree of proteinuria and endothelial dysfunction.

Despite being infrequently examined, *lnc-SSBP2-1:1* showed a high positive correlation with disease activity and superior diagnostic performance (AUC = 1.000). Prior studies have demonstrated that lncRNAs have a role in immunological regulation, specifically through Toll-like receptor signaling, which supports both the innate and adaptive immune responses [34]. Autoimmune illnesses such as rheumatoid arthritis, SLE, and Sjögren’s syndrome are significantly influenced by dysregulated long noncoding RNAs. They can impact the severity and progression of disease by affecting the production of inflammatory cytokines such as TNF-α, IL-6, IL-1β, and IFN-I [35].

Direct mechanistic interplay among these three biomarkers has not been shown in LN. Based on expression profiling, bioinformatics predictions and previous published evidence, there is a potential interplay between them. Our results lend credence to a possible ceRNA-based paradigm. *Lnc-SSBP2-1:1* may function as a molecular sponge for *hsa-miR-126-5p* in this axis, decreasing its capacity to inhibit *TRIM8*. The resulting *TRIM8* derepression may increase NF-κB and type I interferon signaling, which would increase the release of pro-inflammatory cytokines and endothelial dysfunction [36–38]. This interaction highlights the biological relevance of this ncRNA–mRNA network in LN pathogenesis and provides a tenable explanation for the correlations with disease activity that have been found.

Proteinuria, serum creatinine, C3/C4, and anti-dsDNA are current clinical indicators that are not sensitive or specific for disease activity [3]. Our ROC analysis, on the other hand, showed that the TRIM8-associated ncRNA panel outperformed conventional serological markers in terms of discriminatory power (AUCs > 0.93). When combined with well-known clinical indices as SLEDAI-2K, this indicates possible additional value [13, 14].

Compared to kidney biopsy, the use of qPCR-based RNA tests is both technically possible and reasonably priced. These tests could be used as non-invasive supplements to the current LN monitoring techniques if they are properly standardized. Furthermore, TRIM8 inhibition is a possible therapeutic strategy to reduce overactive IFN signaling, albeit these tactics are still in the preclinical stage.

## Conclusion

This work represents one of the earliest investigations into the dysregulated expression of the *TRIM8*-associated non-coding RNA network—encompassing *lnc-SSBP2-1:1* and *hsa-miR-126-5p*—in lupus nephritis. Our findings establish its ability to discriminate between active and inactive disease states and reveal a strong association with disease severity. These results suggest that this RNA panel holds significant promise as both a non-invasive biomarker and a potential therapeutic target. Furthermore, it may complement current monitoring strategies for lupus nephritis; however, rigorous validation in larger and more heterogeneous autoimmune and renal cohorts will be essential to confirm its specificity and advance its clinical applicability.

## Key limitations and future directions

The study has some limitations, including a relatively small sample size and a cross-sectional design, which precludes causal inference or prediction of future flares. Future studies with larger, independent cohorts and mechanistic experiments across diverse renal and autoimmune diseases are needed to confirm the diagnostic and pathogenic roles of *TRIM8*, *miR-126-5p*, and *lnc-SSBP2-1:1* in lupus nephritis.

## Supplementary Information

Below is the link to the electronic supplementary material.

Supplementary Material 1.

## Data Availability

All data supporting the findings of this study are available within the paper, any additional data are available from the corresponding author upon reasonable request.

## Abbreviations

LN: Lupus nephritis
SLE: Systemic lupus erythematosus
mRNA: Messenger ribonucleic acid
ncRNAs: Non-coding RNAs
ESKD: End-stage kidney disease
TRIM8: Tripartite motif-containing protein 8
PCR: Polymerase chain reaction
IFN-I: Type I interferon
NF-κB: Nuclear factor kappa-light-chain-enhancer of activated B cells
miRNAs: MicroRNAs
lncRNAs: Long non-coding RNAs
EULAR/ACR: European league against rheumatism and the american college of rheumatology.
SLEDAI-2K: Systemic lupus erythematosus disease activity index 2000
rSLEDAI: Renal component of the systemic lupus erythematosus disease activity index
CBC: Complete blood count
ANA: Antinuclear antibody
anti-dsDNA: Anti-double-stranded DNA antibodies
C3: Complement 3
C4: Complement 4
CRP: C-reactive protein
BUN: Blood urea nitrogen
CKD-EPI: Chronic kidney disease epidemiology collaboration
NCBI: The national center for biotechnology information
cGAS-STING: Cyclic GMP-AMP synthase-stimulator of interferon genes.
EDTA: Ethylenediaminetetraacetic acid
PBMNCs: Peripheral blood mononuclear cells
AUC: Area under the curve
ROC: Receiver operating characteristic
TNF-α: Tumor necrosis factor alpha
IL: Interleukin
OA: Osteoarthritis
RQ: Relative quantification
eGFR: Estimated glomerular filtration rate
WBC: White blood cells
Pr/Cr ratio: Protein/creatinine ratio
SE: Standard error
CI: Confidence interval
ceRNA: Competing endogenous RNA
