# Supplementary material for: TRIM8-associated non-coding RNA panel as a biomarker for Lupus nephritis activity
Source: J Transl Med. 2025 Nov 5;23:1229. doi: 10.1186/s12967-025-07137-3 (PMC12590613; doi:10.1186/s12967-025-07137-3)
Supplement: Supplementary file 1 — Supplementary Material 1. [file 12967_2025_7137_MOESM1_ESM.docx]

**Supp. Figure 1. Genetic Network Selection**


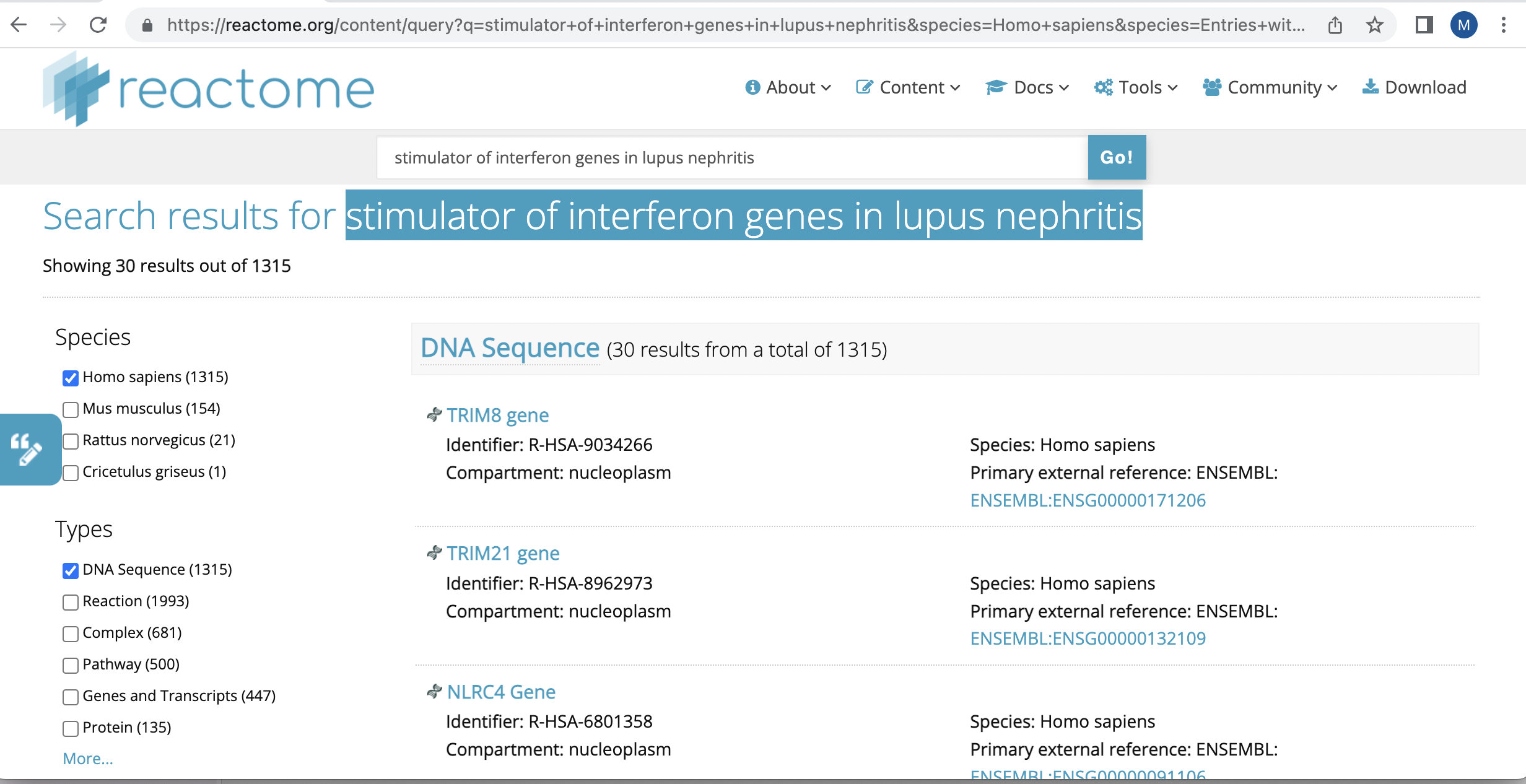


**A.** Snapshot shows the involvement of genes related to the interferon stimulator gene pathway in LN using the Reactome database (<https://reactome.org/>)


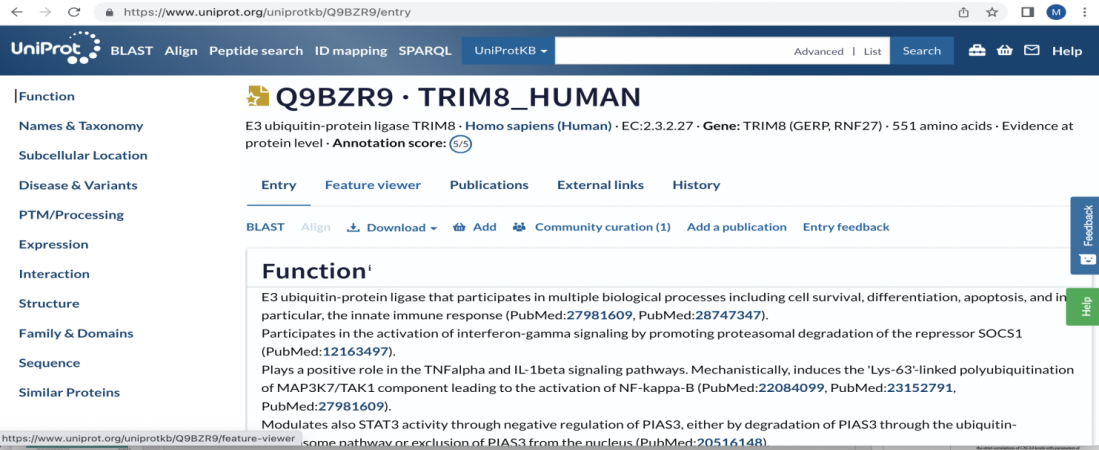


**B.** Snapshot shows verification of the function of gene of interest (*TRIM8*) and its involvement in the Interferon response pathway using Uniprot database (<https://www.uniprot.org/>)


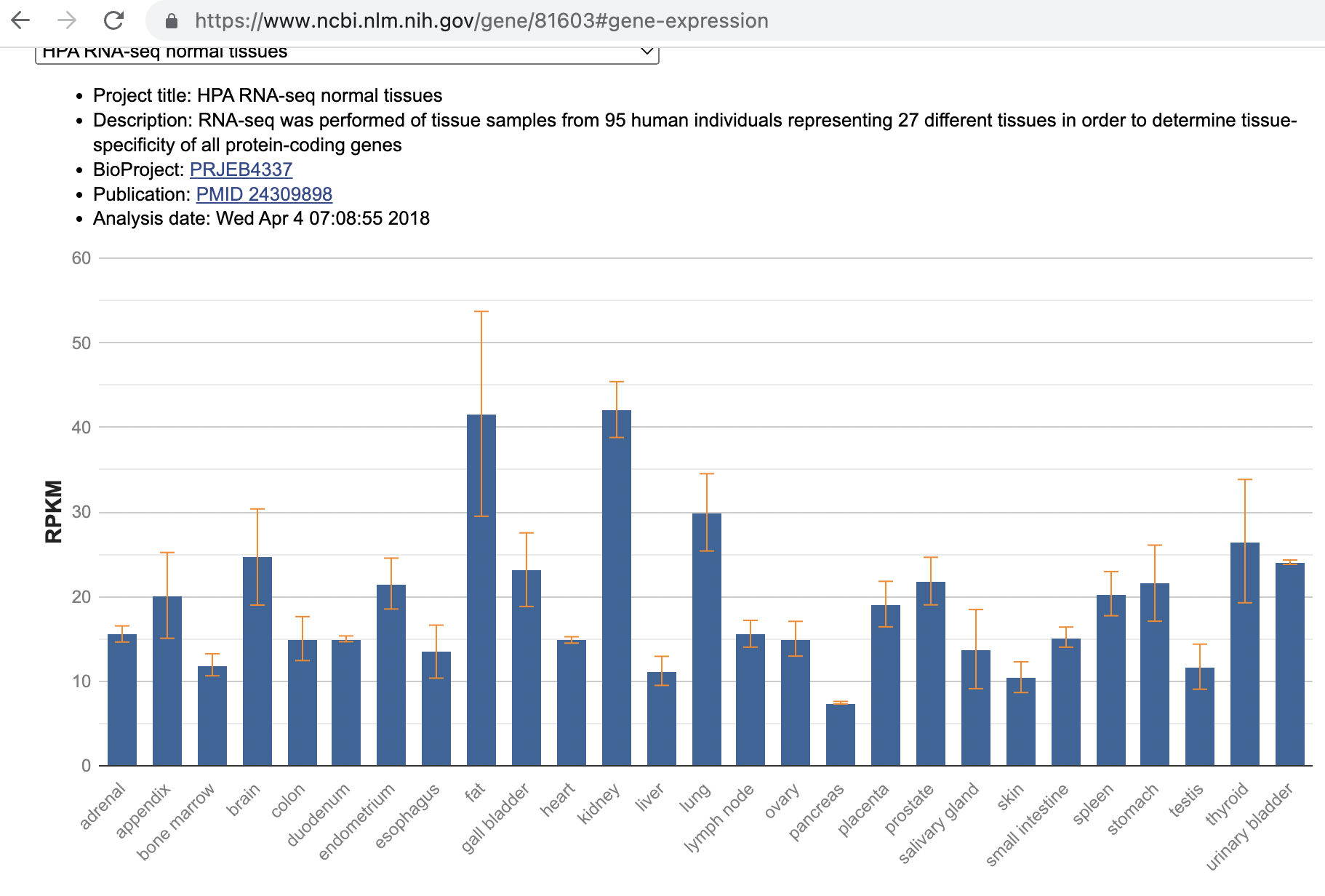


**C.** Snapshot shows high expression of *TRIM8* in the kidney using NCBI database (<https://www.ncbi.nlm.nih.gov/>)


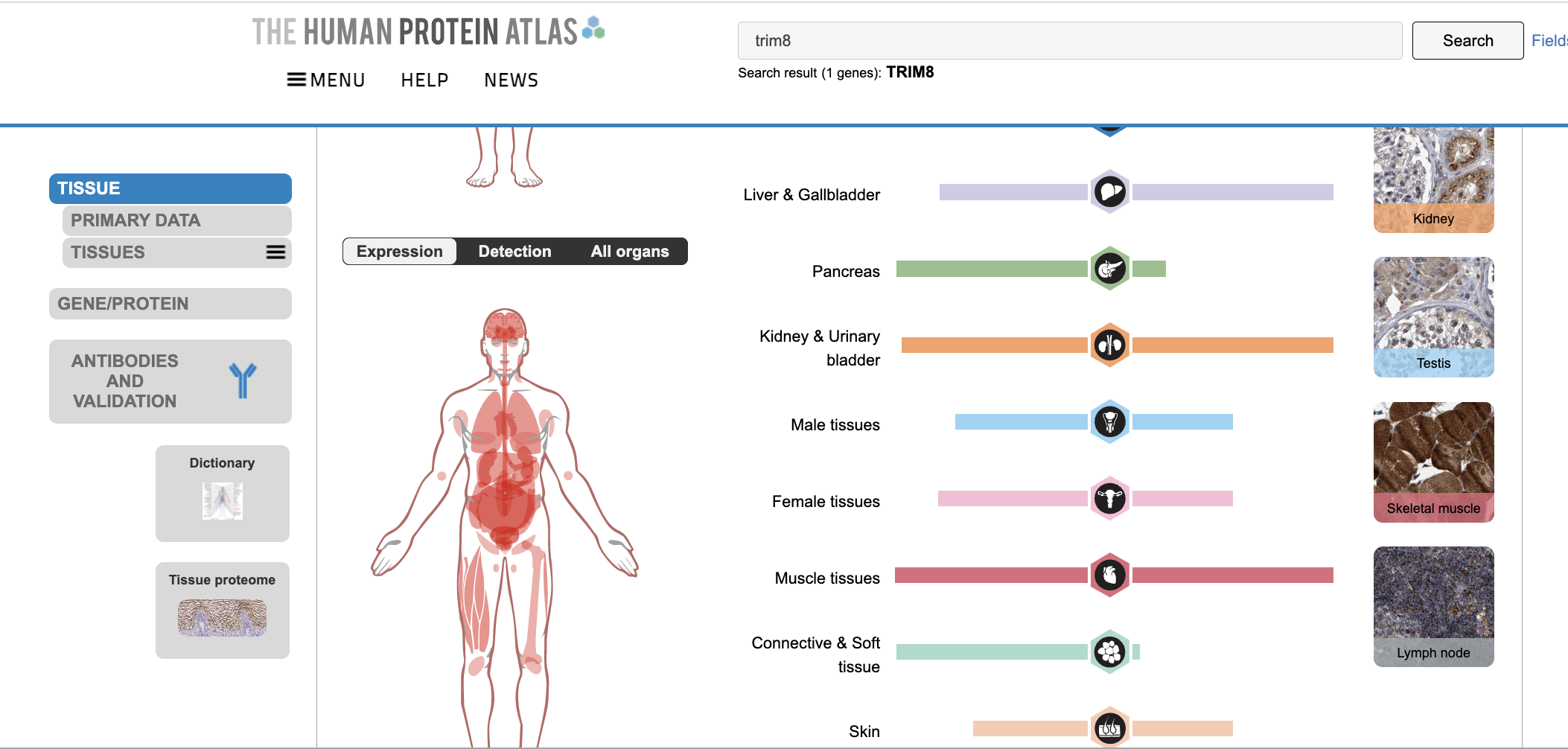


**D.** Snapshot shows expression of *TRIM8* in tissue of interest (kidney) from protein atlas database (<https://www.proteinatlas.org/>)


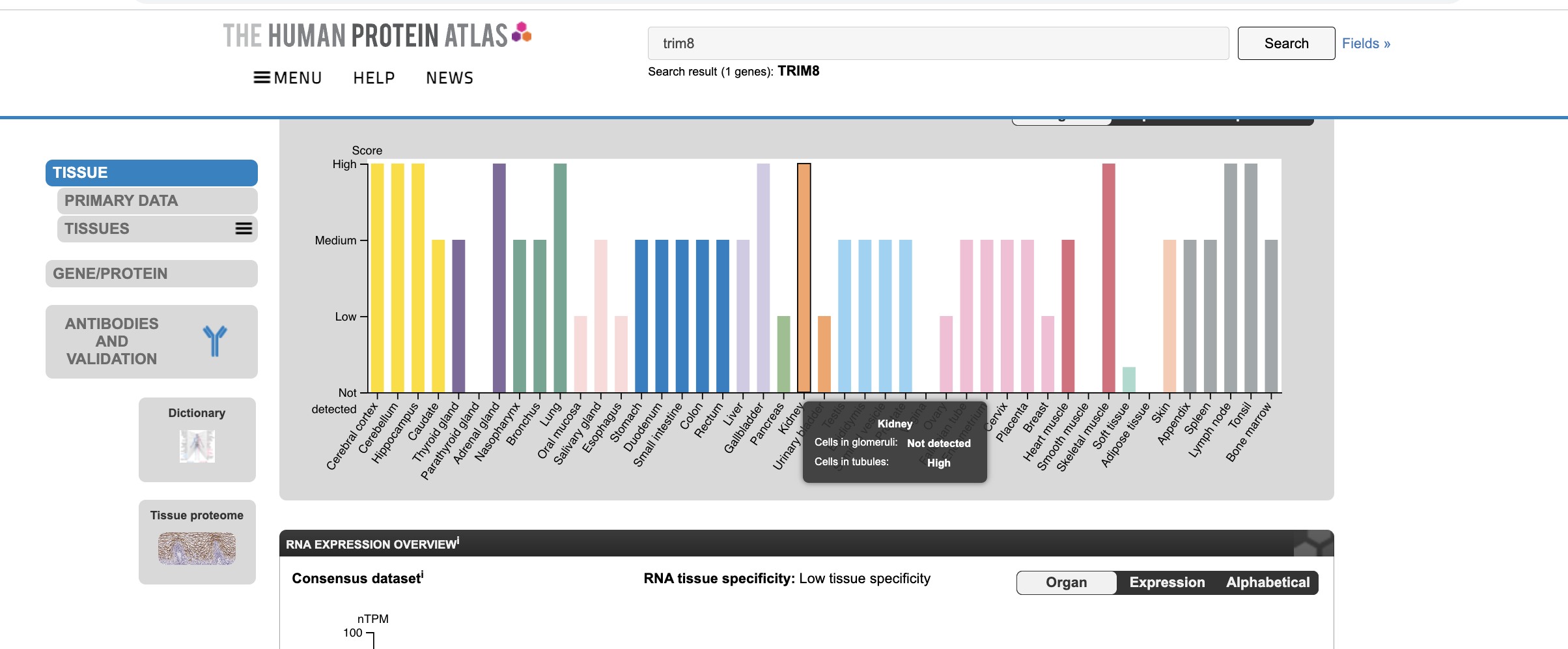


**E.** Snapshot of *TRIM8* gene expression in kidneys from protein atlas database (<https://www.proteinatlas.org/>)


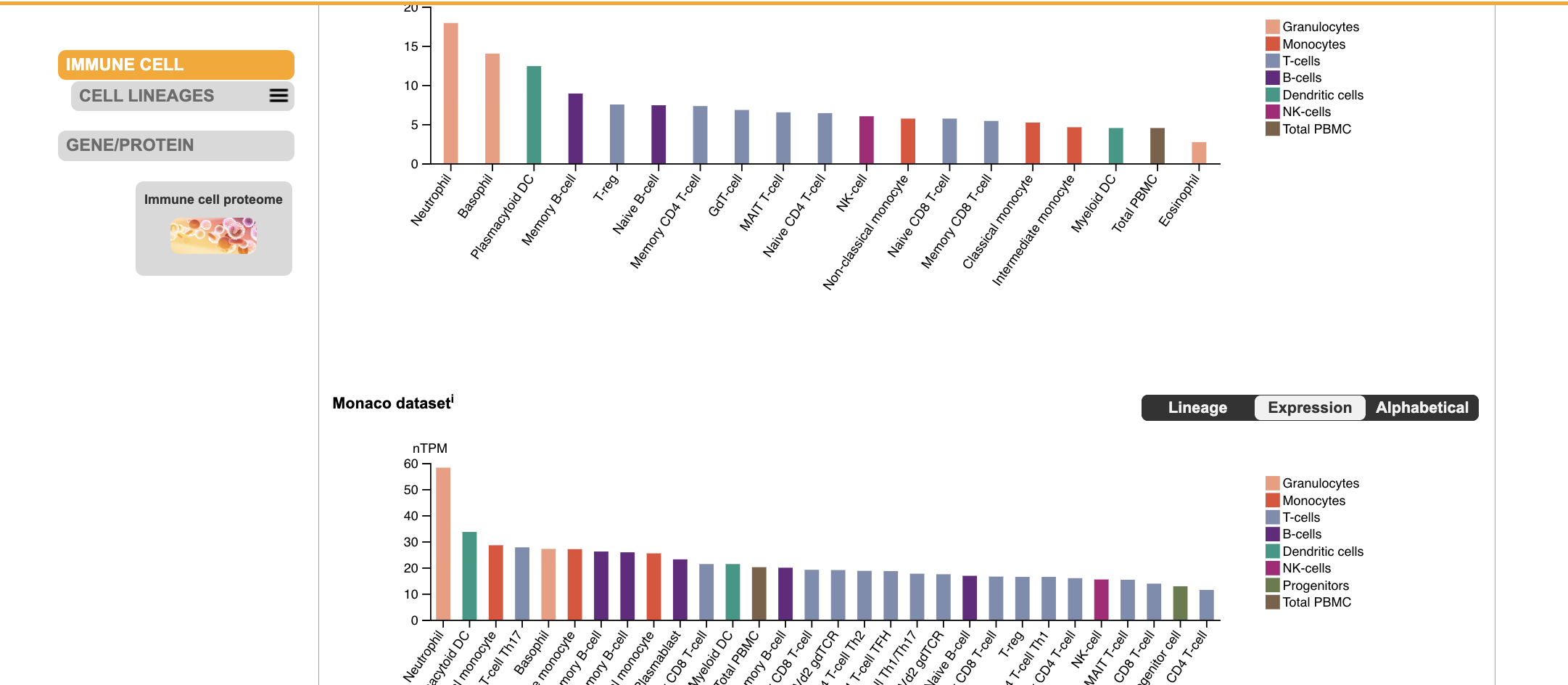


**F.** Snapshot shows the *TRIM8* Gene expression in WBC components in blood using protein atlas database (<https://www.proteinatlas.org/>)


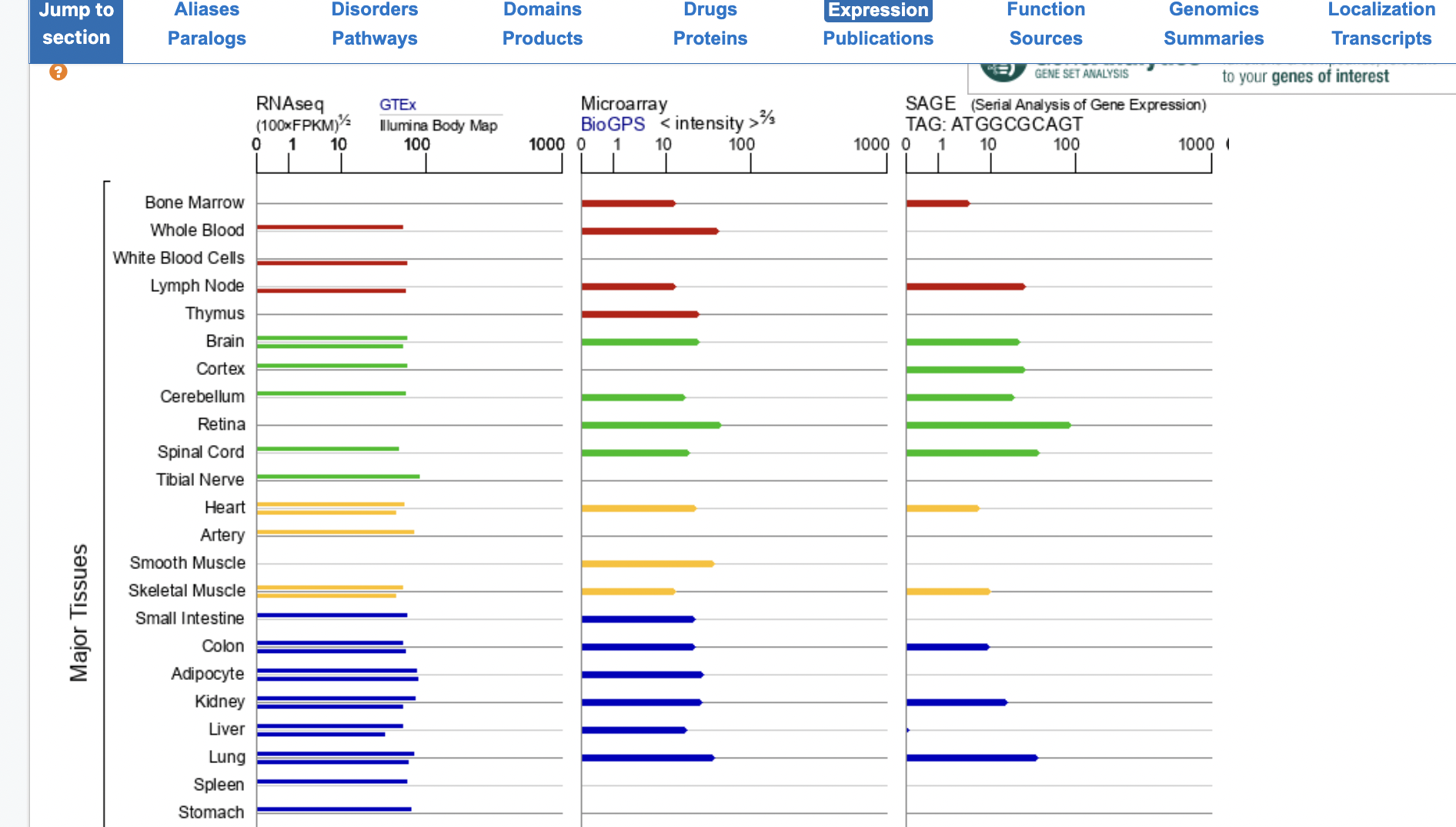


**G.** Snapshot shows *TRIM8* Gene expression in WBCs using Gene Cards database (<https://www.genecards.org/>)

**Supp. Figure 2. Selection of epigenetic regulators**


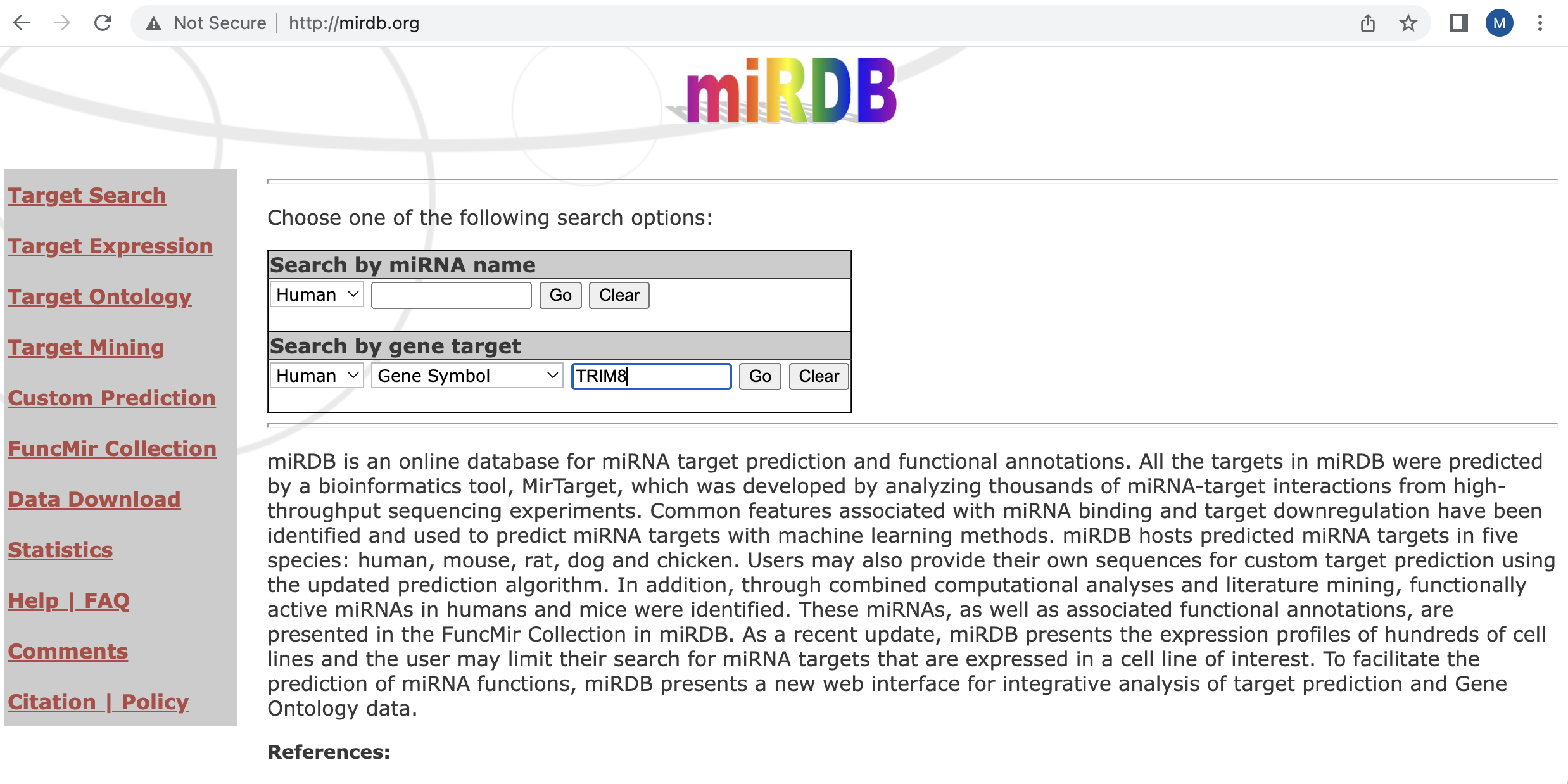


**A.** Snapshot shows the retrieval of (*hsa-miR126-5p*) miRNA related to the selected gene (*TRIM8*) using mirDB database (<https://mirdb.org/>)


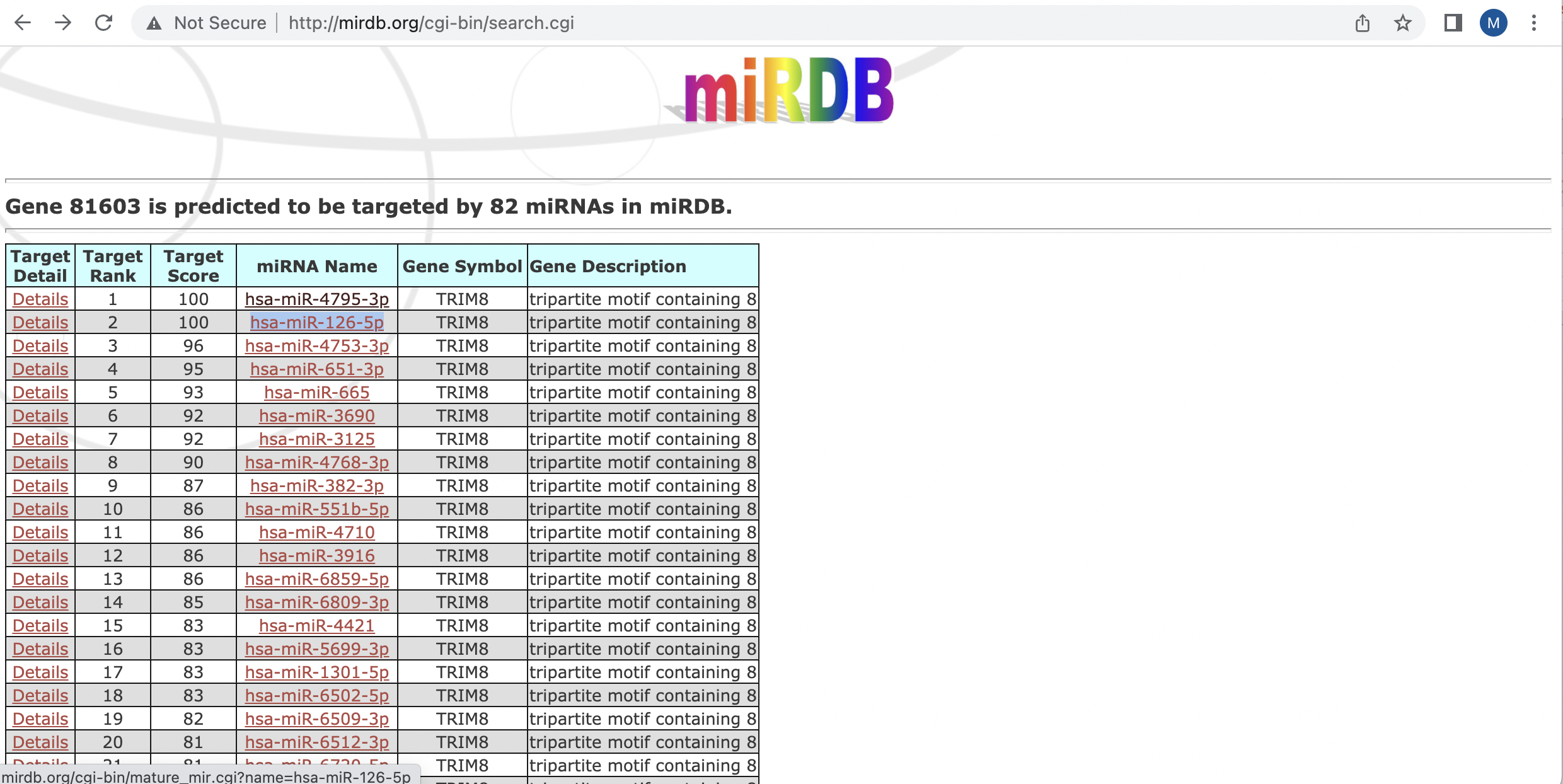


**B.** Snapshot shows the retrieval of (*hsa-miR126-5p*) miRNA related to the selected gene (*TRIM8*) with high target score using mirDB database (<https://mirdb.org/>)


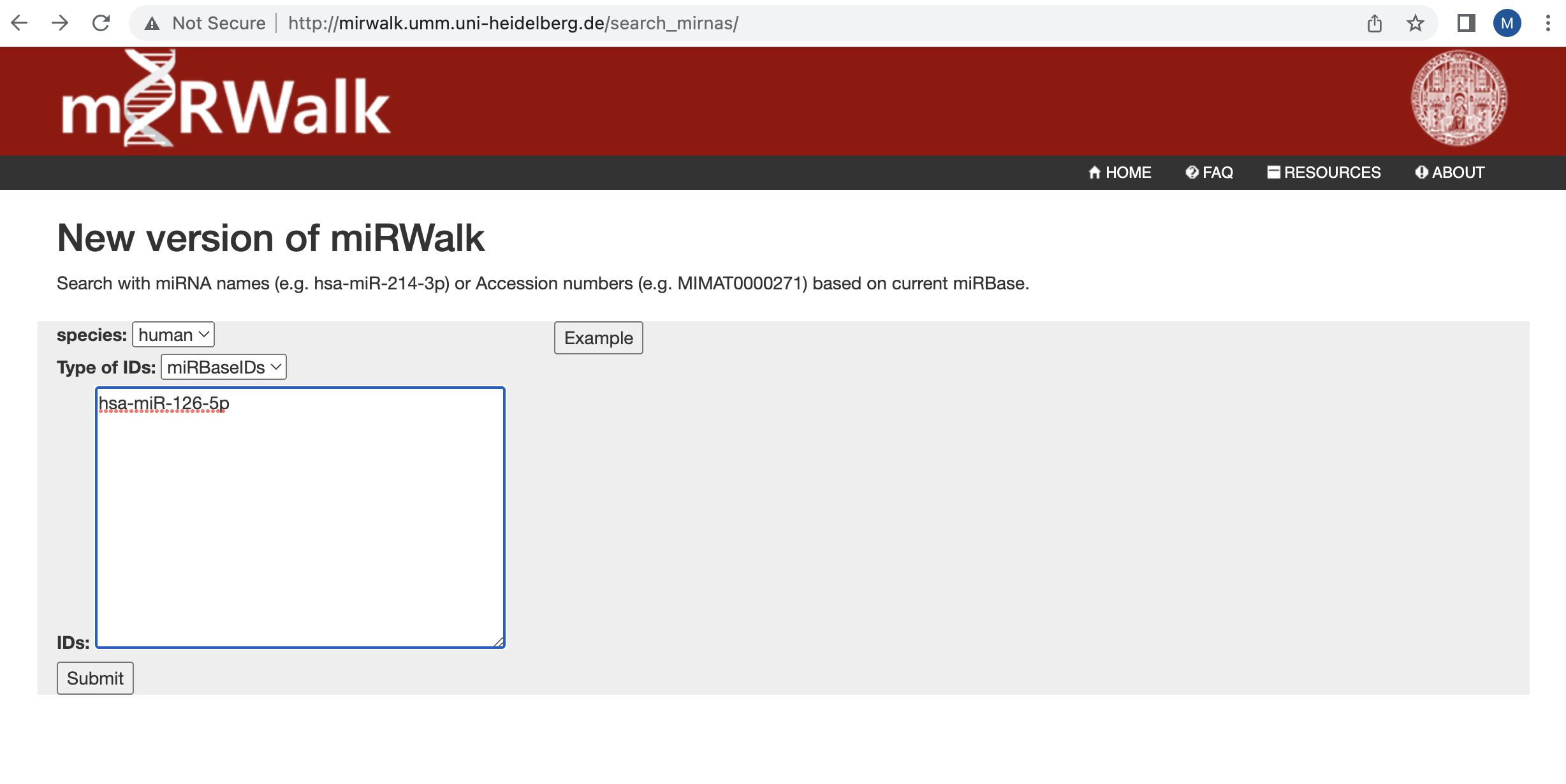


**C.** Snapshot shows the retrieval of lncRNA (*lnc-SSBP2-1:1*) related to the selected miRNA (*hsa-miR126-5p*) using mirwalk database. (<http://mirwalk.umm.uni-heidelberg.de/>)


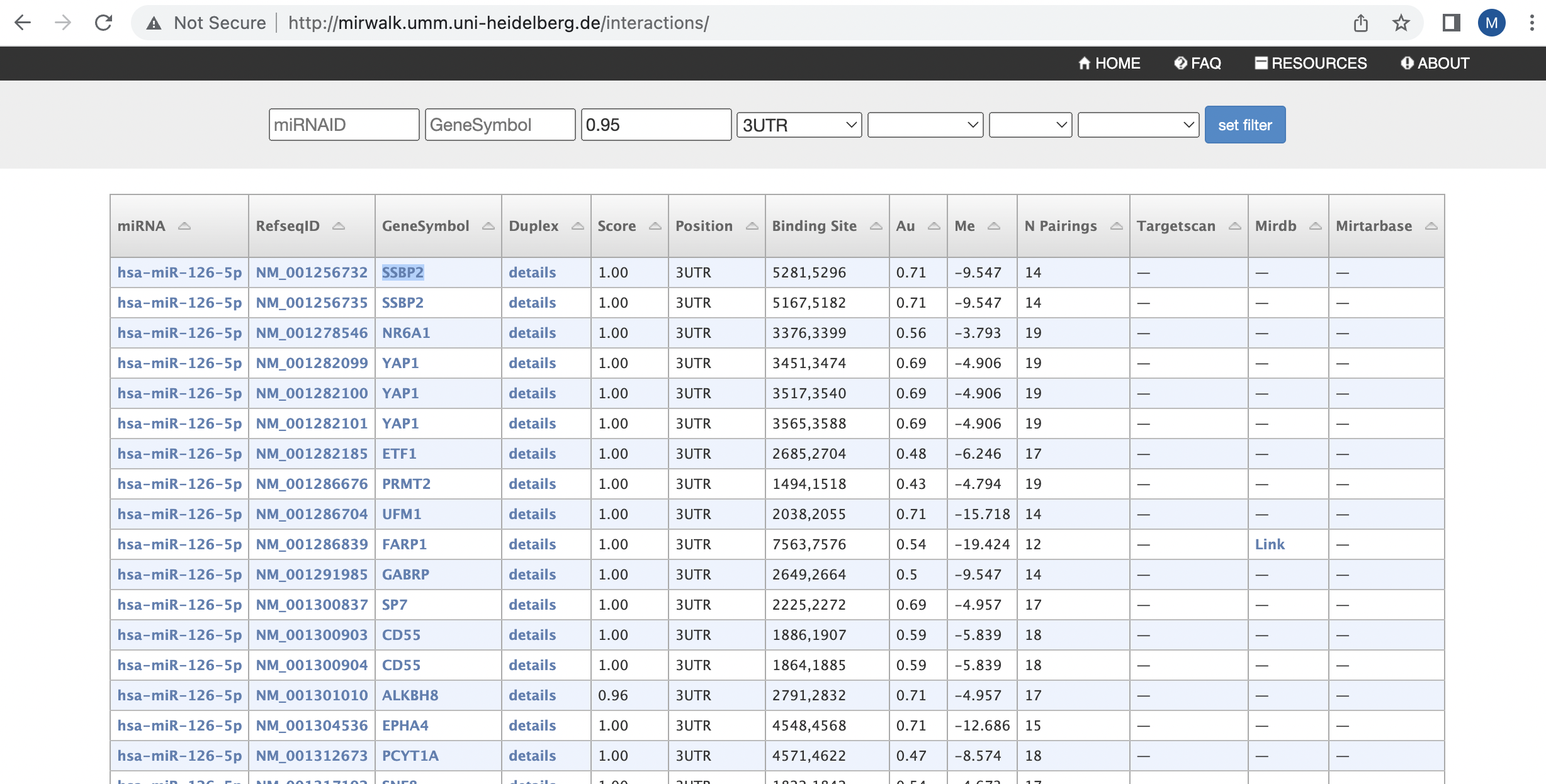


**D.** Snapshot shows the retrieval of lncRNA (*lnc-SSBP2-1:1*) related to the selected miRNA (*hsa-miR126-5p*) using mirwalk database (<http://mirwalk.umm.uni-heidelberg.de/>)

**Supp. Figure 3. Receiver operating characteristic (ROC) curves of traditional biomarkers (serum creatinine, complement C3, and complement C4) for differentiating active from inactive lupus nephritis.**


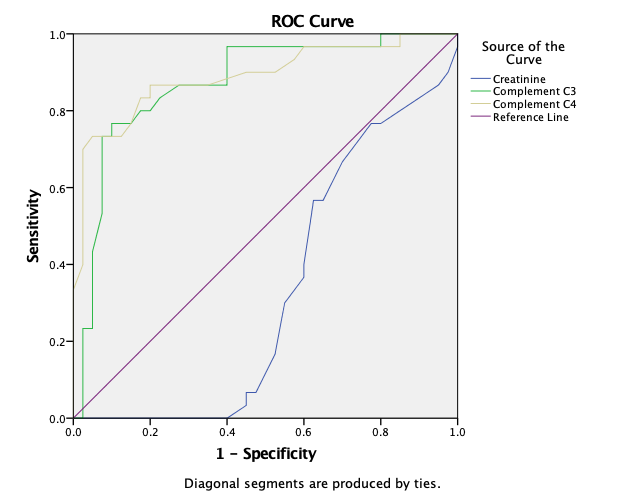


**Supp. Table 1. Comparison between the active and the non-active LN groups about Demographic and clinical characteristics:**

| **Variables** | | | **Active lupus nephritis**  **(Total=40)** | **Non-active lupus nephritis**  **(Total=30)** | **p-value** |
| --- | --- | --- | --- | --- | --- |
| **Age**  **(years)** | **Mean ± SD** | | 26.7±8.2 | 28.6±6.9 | ^0.384 |
| **Gender**  **(n, %)** | **Male** | | 12 (30.0%) | 4 (13.3%) | #0.259 |
|  | **Female** | | 28 (70.0%) | 26 (86.7%) |  |
| **Duration of SLE**  **disease (years)** | | **Median** | 2.0 (0.5–5.0) | 6.0 (3.8–9.0) | **^<0.001*** |
| **SLEDAI 2K score** | | **Median** | 18.0 (12.0–20.0) | 4.0 (3.5–8.3) | **^<0.001*** |
| **SLEDAI renal score** | | **Median** | 12.0 (8.0–12.0) | 0.0 (0.0–0.0) | **^<0.001*** |
| **Hypertension** | | | 18 (45.0%) | 5 (16.7%) | **#****0.013*** |
| **Anti-phospholipid syndrome** | | | 6 (15.0%) | 8 (26.7%) | #0.227 |

^ANOVA test. ^Mann-Whitney test. §Fisher’s Exact test. # Chi-square test. *Significant p-value < 0.05.

**Supp. Table 2. Correlations of RQ of *TRIM8-*associated ncRNA regulatory network expressions in the active LN group (n=40):**

| **Variables** | | ***TRIM-8* mRNA RQ** | | ***hsa-miR-126-5p* miRNA RQ** | | ***lnc-SSBP2-1:1***  **lncRNA RQ** | |
| --- | --- | --- | --- | --- | --- | --- | --- |
|  |  | **r** | **p-value** | **r** | **p-value** | **R** | **p-value** |
| **^Age (years)** | | 0.027 | 0.869 | -0.149 | 0.360 | -0.091 | 0.576 |
| **#Duration of SLE disease** | | -0.221 | 0.172 | -0.037 | 0.819 | -0.353 | **0.026*** |
| **#SLEDAI 2K score** | | 0.161 | 0.321 | 0.055 | 0.737 | -0.177 | 0.276 |
| **#SLEDAI renal score** | | -0.021 | 0.897 | 0.043 | 0.793 | 0.025 | 0.879 |
| **^Hemoglobin (gm/dL)** | | -0.300 | 0.060 | -0.090 | 0.582 | 0.028 | 0.866 |
| **^WBC (x10^3^/mL)** | | 0.002 | 0.988 | -0.213 | 0.188 | -0.139 | 0.393 |
| **^Platelets (x10^3^/mL)** | | -0.025 | 0.877 | 0.070 | 0.669 | 0.001 | 0.995 |
| **^Serum albumin (gm/dL)** | | -0.153 | 0.345 | -0.521 | **0.001*** | -0.085 | 0.602 |
| **^Serum creatinine (mg/dL)** | | 0.058 | 0.720 | -0.182 | 0.260 | -0.073 | 0.654 |
| **^Serum BUN (mg/dL)** | | 0.125 | 0.443 | -0.218 | 0.176 | -0.162 | 0.317 |
| **^eGFR (mL/min/1.73m^2^)** | | 0.016 | 0.923 | 0.156 | 0.338 | 0.064 | 0.693 |
| **^CRP (mg/dL)** | | -0.049 | 0.763 | -0.087 | 0.594 | -0.023 | 0.889 |
| **^C3 (mg/dL)** | | -0.247 | 0.124 | -0.189 | 0.242 | 0.103 | 0.527 |
| **^C4 (mg/dL)** | | -0.251 | 0.118 | -0.113 | 0.486 | 0.233 | 0.148 |
| **^ANA titre** | | 0.057 | 0.729 | 0.079 | 0.631 | -0.168 | 0.306 |
| **^Pr/Cr ratio (gm/gm)** | | -0.106 | 0.515 | 0.045 | 0.782 | -0.001 | 0.996 |
| **Anti-DNA** | **Yes** | 366.6±301.8 | | 0.02±0.02 | | 37602.9±55652.5 | |
|  | **No** | 192.9±118.7 | | 0.01±0.01 | | 18195.2±14689.2 | |
|  | **p-value** | **0.013*** | | **0.038*** | | 0.264 | |

Total=40. ^Pearson test. #Spearman test. ^Independent t-test. #ANOVA test. *Significant p-value <0.05.

**Supp. Table 3. Correlations of RQ of *TRIM8-*associated ncRNA regulatory network expressions in the non-active LN group (n=30):**

| **Variables** | ***TRIM-8* mRNA RQ** | | ***hsa-miR-126-5p* miRNA RQ** | | ***lnc-SSBP2-1:1***  **lncRNA RQ** | |
| --- | --- | --- | --- | --- | --- | --- |
|  | **r** | **p-value** | **r** | **p-value** | **r** | **p-value** |
| **^Age (years)** | 0.198 | 0.294 | -0.126 | 0.507 | 0.111 | 0.560 |
| **#Duration of SLE disease (years)** | 0.258 | 0.169 | -0.047 | 0.806 | 0.057 | 0.765 |
| **#SLEDAI 2K score** | 0.088 | 0.643 | 0.108 | 0.569 | -0.168 | 0.374 |
| **^Hemoglobin (gm/dL)** | -0.036 | 0.850 | -0.132 | 0.487 | 0.212 | 0.261 |
| **^WBC (x10^3^/mL)** | -0.132 | 0.486 | -0.133 | 0.485 | 0.016 | 0.932 |
| **^Platelets (x10^3^/mL)** | 0.010 | 0.958 | -0.065 | 0.736 | -0.294 | 0.122 |
| **^Serum albumin (gm/dL)** | 0.053 | 0.780 | -0.121 | 0.523 | -0.077 | 0.686 |
| **^Serum creatinine (mg/dL)** | -0.030 | 0.877 | -0.230 | 0.222 | 0.147 | 0.439 |
| **^Serum BUN (mg/dL)** | 0.015 | 0.939 | -0.022 | 0.906 | -0.126 | 0.506 |
| **^eGFR (mL/min/1.73m^2^)** | -0.066 | 0.728 | 0.200 | 0.289 | -0.176 | 0.353 |
| **^CRP (mg/dL)** | 0.137 | 0.471 | 0.352 | 0.057 | -0.288 | 0.123 |
| **^C3 (mg/dL)** | -0.243 | 0.195 | -0.320 | 0.085 | 0.336 | 0.069 |
| **^C4 (mg/dL)** | -0.003 | 0.987 | -0.348 | 0.060 | 0.265 | 0.157 |
| **^ANA titre** | 0.315 | 0.090 | 0.185 | 0.328 | -0.437 | **0.016*** |
| **^Pr/Cr ratio (gm/gm)** | -0.040 | 0.833 | 0.027 | 0.888 | -0.083 | 0.665 |

Total=30. ^Pearson test. #Spearman test. *Significant

**Supp. Table 4. Relation of RQ of *TRIM8-*associated ncRNA regulatory network expressions with gender, hypertension, and antiphospholipid in the active and non-active LN groups**

| **Variables** | | **Active LN group** | | | **non-active LN group** | | |
| --- | --- | --- | --- | --- | --- | --- | --- |
|  |  | ***TRIM-8* mRNA RQ** | ***hsa-miR-126-5p* miRNA RQ** | ***lnc-SSBP2-1:1* lncRNA RQ** | ***TRIM-8* mRNA RQ** | ***hsa-miR-126-5p* miRNA RQ** | ***lnc-SSBP2-1:1* lncRNA RQ** |
| **Gender** | **Male** | 410.2±373.8 | 0.02±0.02 | 19588.6±18073.4 | 18.8±3.5 | 1.25±1.81 | 41.6±19.1 |
|  | **Female** | 279.7±215.3 | 0.01±0.02 | 37698.9±56284.7 | 17.5±13.0 | 0.59±1.41 | 50.7±34.8 |
|  | **p-value** | 0.171 | 0.342 | 0.285 | 0.846 | 0.408 | 0.616 |
| **Hypertension** | **Yes** | 326.2±292.6 | 0.02±0.02 | 26148.9±56630.9 | 32.3±15.0 | 1.75±3.11 | 35.6±27.0 |
|  | **No** | 312.9±265.0 | 0.02±0.02 | 37270.5±41483.5 | 14.8±9.3 | 0.46±0.80 | 52.3±33.9 |
|  | **p-value** | 0.881 | 0.708 | 0.478 | **0.002*** | 0.070 | 0.310 |
| **Antiphospholipid**  **Syndrome** | **Yes** | 420.2±312.1 | 0.02±0.03 | 50418.5±97784.7 | 23.3±12.8 | 1.41±2.41 | 37.5±29.8 |
|  | **No** | 301.0±268.1 | 0.02±0.02 | 29062.4±35568.1 | 15.7±11.5 | 0.41±0.83 | 53.8±33.7 |
|  | **p-value** | 0.333 | 0.722 | 0.327 | 0.130 | 0.092 | 0.239 |

^Independent t-test. *Significant p-value <0.05.
